# Laryngeal preservation in stage III/IV resectable laryngo-hypopharyngeal squamous cell carcinoma following concurrent chemoradiotherapy with capecitabine/cisplatin

**DOI:** 10.3892/mco.2013.113

**Published:** 2013-05-09

**Authors:** DONGBIN AHN, JAE HYUG KIM, JIN HO SOHN, CHANG-MIN SIN, JEONG EUN LEE

**Affiliations:** 1 Departments of Otorhinolaryngology-Head and Neck Surgery, Kyungpook National University, Daegu 700-712, Republic of Korea; 2 Radiation Oncology, School of Medicine, Kyungpook National University, Daegu 700-712, Republic of Korea

**Keywords:** head and neck cancer, chemoradiotherapy, organ preservation, cisplatin, capecitabine

## Abstract

Concurrent chemoradiotherapy has become the standard of care for advanced head and neck cancers in organ preservation strategies. However, the optimal regimen for concurrent chemoradiotherapy to maximise treatment response and minimise toxicity has not been determined. The purpose of the present clinical study was to review our experience with concurrent chemoradiotherapy by using the capecitabine and cisplatin (XP) regimen with the aim of organ preservation in patients with locoregionally advanced laryngo-hypopharyngeal squamous cell carcinoma (LHSCC). Thirty-one patients with stage III/IV resectable LHSCC treated with concurrent chemoradiotherapy using the XP regimen were enrolled in the present study. The patients fulfilled the criteria for total laryngectomy when surgery was selected. The primary endpoint of this study was disease-free survival with a functional larynx, which was indicated by the following conditions: survival, disease-free status at all sites and retention of a functional larynx. Following concurrent chemoradiotherapy, 23 patients (74.2%) exhibited complete response (CR) at the primary site and 18 (69.2%) exhibited CR at the lymph nodes. CR at the primary site and lymph nodes was identified in 19 patients (61.3%). The Kaplan-Meier 2- and 3-year cumulative disease-free survival rates were 71.5 and 59.6%, respectively. Anatomical laryngeal preservation was feasible in 27 patients (87.1%) during the 36-month follow-up period. Permanent gastrostomy was required in 1 disease-free patient with an intact larynx. For the entire cohort, the 2- and 3-year cumulative disease-free survival with a functional larynx was 58.5 and 50.7%, respectively. The most frequent grade 3–4 haematological and non-haematological toxicities of concurrent chemoradiotherapy were leucopenia and mucositis, which developed in 4 (12.9%) and 8 (25.8%) patients, respectively. There was no treatment-related death. Concurrent chemoradiotherapy with the XP regimen resulted in functional laryngeal preservation accompanied by disease-free survival and the toxicities were more tolerable and manageable compared to those reported by previous studies.

## Introduction

Over several decades, surgery has been the standard treatment selection for squamous cell carcinoma (SCC) of the head and neck. However, as concerns regarding quality of life and organ preservation have recently been raised, the effect of treatment on functions such as speech and eating have become critical factors for selecting a treatment modality. Therefore, studies have focused on organ preservation strategies to achieve disease control and survival and maintain speech and swallowing functions (1–3).

The Radiation Therapy Oncology Group (RTOG) 91-11 trial suggested concurrent chemoradiotherapy as the standard treatment for laryngeal preservation in patients with locoregionally advanced laryngeal cancer (4,5). In addition, according to a large meta-analysis, concurrent chemoradiotherapy had the best survival outcome among all the regimens of chemotherapy and radiotherapy that were assessed (3). Consequently, the use of concurrent administration of chemotherapy and radiotherapy has become the standard of care for advanced head and neck cancers in organ preservation strategies. However, the optimal regimen for concurrent chemoradiotherapy to maximise treatment response and minimise toxicity, excluding the distinct evidence regarding the use of cisplatin-based regimens, has not been determined.

The combination of cisplatin and infusional 5-fluorouracil (5-FU), which is known as the FP regimen, has been widely used as a standard regimen in concurrent chemoradiotherapy for squamous cell carcinoma of the head and neck, with a response rate of ∼40–80% reported by previous studies (6,7). Despite considerable experience with this regimen, 5-FU is administered as a 5-day continuous infusion to promote its activity and enhance the response rate to cisplatin. Continuous infusion via intravenous access devices required by the FP regimen may cause several complications, such as catheter-related infection and thrombosis, thus decreasing treatment compliance and causing deterioration in the quality of life of the patient during treatment. Furthermore, additional treatment-related costs are incurred.

Capecitabine (Xeloda^®^; Hoffmann-La Roche Inc., Nutley, NJ, USA) is a 5-FU prodrug characterised by a pyrimidine ring with a fluorine atom at the 5′ position. Following oral administration, it crosses the gastrointestinal barrier intact and is rapidly and almost completely absorbed. It is subsequently activated into 5-FU, preferentially in tumours. Previous studies reported that the efficacy of capecitabine is similar to that of 5-FU in advanced colorectal and breast cancer (8–10) and the US Food and Drug Administration has approved the administration of capecitabine to patients with advanced breast and colorectal cancer. However, the efficacy of capecitabine treatment in head and neck cancer has not yet been determined. Previously, we reported the results of a phase II study of concurrent chemoradiotherapy with capecitabine and cisplatin (XP regimen) in squamous cell carcinoma of the head and neck, in which we observed a 94.6% overall response rate [complete response (CR), 78.4%; partial response (PR), 16.2%] (1). We have since then used the capecitabine/cisplatin combination as an initial chemotherapy regimen when concurrent chemoradiotherapy was performed for squamous cell carcinoma of the head and neck to improve survival and organ preservation and decrease toxicity.

The purpose of the present clinical study was to review our experience with concurrent chemoradiotherapy using XP and evaluate the efficacy and safety of the XP regimen with regard to organ preservation in patients with advanced laryngo-hypopharyngeal squamous cell carcinoma (LHSCC).

## Materials and methods

### Patient population

A database of patients with head and neck cancer was established upon the inception of the Head and Neck Cancer Centre of our institution in 1997. Using this database, we reviewed the medical records of patients who were treated with concurrent chemoradiotherapy as the primary treatment for histologically confirmed LHSCC between January, 2004 and March, 2010.

The patients were required to meet the following conditions to receive concurrent chemoradiotherapy: a performance status score of 0–2 on the Eastern Cooperative Oncology Group scale and a life expectancy of >12 weeks. In addition, we imposed the following requirements: adequate haematological [white blood cell count (WBC) ≥4000/*μ*l; haemoglobin ≥10 g/dl; and platelet count ≥100,000/*μ*l], renal (serum creatinine ≤1.5 mg/dl and creatinine clearance ≥50 ml/min) and hepatic (total bilirubin ≤2.0 mg/dl and serum transaminase levels ≤3 times the upper limit of the normal range) function; no history of seizures; no central nervous system or psychiatric disorders that may alter treatment compliance; no clinically significant cardiac disease; and no serious uncontrolled infections.

Patients were excluded from the study if they had any history of laryngo-hypopharyngeal malignancy related to recurrent disease and previous surgery, irradiation, or chemotherapy for other squamous cell carcinomas of the head and neck. Patients whose medical records or medical charts were inaccessible were also excluded. The Institutional Review Board of our institution approved the treatment regimens employed in this study (registration no. 74005-2444). Written informed consent was obtained from the patients prior to treatment.

### Treatment plan

In the XP regimen, the method of administration was essentially identical to that of the previously reported protocol, except for the cisplatin dose, which was reduced from 80 to 60 mg/m^2^(1) since, in our experience, the majority of the critical complications from concurrent chemoradiotherapy with cisplatin and capecitabine are associated with cisplatin rather than capecitabine. Cisplatin was administered as a 1-h intravenous infusion on the first day of each cycle, followed by oral capecitabine (825 mg/m^2^ twice daily) with oral pyridoxine (100 mg three times a day) on days 1–14 and a 7-day rest period on days 15–21. Oral ondansetron hydrochloride (8 mg) was provided every 12 h as a prophylactic antiemetic drug during capecitabine administration and an additional 5 mg of oral metoclopramide hydrochloride was administered as needed for symptomatic nausea and/or vomiting. Capecitabine was administered as film-coated tablets of two dose strengths (150 and 500 mg): if the patients were unable to swallow a tablet, capecitabine was dissolved in water or administered through a feeding tube.

Megavoltage X-ray generated by a 6-MV linear accelerator was used in radiotherapy. Radiotherapy was initiated on day 1 of the concurrent chemoradiotherapy schedule: 1.8–2.0 Gy/day was administered to the primary site and neck metastasis for 5 days a week, until a total dose of 66–72 Gy. Elective neck irradiation up to 45–50 Gy was applied to tumour-free areas when indicated. Generally, two cycles of chemotherapy were repeated every 21 days during concurrent radiotherapy. An additional cycle of chemotherapy was administered on an individual basis according to the response to concurrent chemoradiotherapy.

### Assessment of toxicity and schedule modification

Patients were monitored for toxicity and adverse effects on the basis of their medical history and physical examination, as well as laboratory studies, including complete blood counts and serum biochemistry tests, throughout the duration of the concurrent chemoradiotherapy. Toxicities were recorded at each visit (once a week during concurrent chemoradiotherapy) and graded according to the National Cancer Institute Common Toxicity Criteria version 3.0 until the end of the last cycle of chemotherapy. If grade 3 or 4 chemotherapy-related haematological or non-haematological toxicity was observed, capecitabine was withheld until the toxicity was resolved to the baseline or less than grade 1. Capecitabine was resumed at the original dose level after recovery in such cases. If myelosuppression (WBC <4000/*μ*l or haemoglobin <10 mg/dl) was detected after the completion of each cycle of chemotherapy, the next cycle was postponed for 1 week.

### Assessment of treatment response

Treatment response was assessed by clinical examination, including laryngoscopic examination and computed tomography, 8 weeks after the completion of the concurrent chemoradiotherapy. Biopsy or fine-needle aspiration cytology was not routinely performed to evaluate the pathologic response. However, positron emission tomography/computed tomography and cervical computed tomography with contrast enhancement were routinely performed. Responses were defined as CR, PR, stable disease (SD) and progressive disease (PD), according to the criteria requirements of the World Health Organization (11).

### Salvage treatment

Salvage surgery was recommended for the patients who failed to achieve CR following completion of concurrent chemoradiotherapy, provided that curative surgical management of the residual disease was possible. Surgery was generally performed 8–10 weeks after concurrent chemoradiotherapy. In cases of non-CR at the primary site, total laryngectomy with partial pharyngectomy was usually recommended, as the patients without CR at the primary site had advanced disease. For patients who did not achieve CR at the cervical lymph nodes, the extent of surgery ranged from a wide excisional biopsy to a comprehensive neck dissection.

### Follow-up strategy for patients with CR

Patients who achieved a CR were advised to visit on a monthly basis for at least the first 6 months, then every other month for the following 6 months. If there was no evidence of disease after 1 year of follow-up, the patient visited every third month during the second year of follow-up. A 6-month follow-up interval was allowed thereafter. Clinical evaluation, including laryngoscopic examination, was performed at every visit. Additionally, plain chest radiographs and laboratory tests, including complete blood counts, serum biochemistry and thyroid function tests were performed annually. The patients underwent a positron emission tomography/computed tomography scan every year as a screening study tool to detect recurrent disease and second primary malignancy.

### Statistical analyses

The primary endpoint of this study was disease-free survival with a functional larynx, which was estimated from the date of treatment initiation using the Kaplan-Meier method. For this endpoint, events were defined as death from any cause, locoregional/distant progression or relapse, tracheotomy, feeding tube insertion, gastrostomy or laryngectomy, depending on which event occurred first. Disease-free status was estimated by considering persistent or recurrent disease at the primary site, adjacent non-nodal structures, cervical lymph nodes and/or distant organs after concurrent chemoradiotherapy as an event. The secondary endpoints were disease-free survival and the pattern of treatment failure such as locoregional progression or relapse. Patients who did not experience an event were censored on the last date that they were known to be alive. The time-to-event curves of these endpoints were obtained by the Kaplan-Meier method using SPSS software for Windows version 12.0 (SPSS, Inc., Chicago, IL, USA).

## Results

### Patient characteristics and TNM stage

Between January, 2004 and March, 2010, 75 patients with LHSCC initially treated with concurrent chemoradiotherapy were identified in our institution. Among these, 31 patients with stage III/IV resectable LHSCC treated with concurrent chemoradiotherapy using the XP regimen were eligible for enrollment in our study. All the patients fulfilled the criteria for total laryngectomy when surgery was selected.

Of the 75 patients, 12 (16.0%) were treated with concurrent chemoradiotherapy using the FP regimen and 4 (5.3%) had unresectable LHSCC encasing the carotid artery by >270° or with distant metastasis at initial diagnosis. Twenty-five patients (33.3%) had early stage or T3 disease that did not require total laryngectomy. These patients were excluded from our study. The remaining 3 patients (4.0%) were also excluded due to inadequate medical records.

The patient characteristics and TNM stage are summarised in Table I. The cohort of 31 patients comprised 29 men and 2 women, with a mean age of 66.3±7.6 years at the time of initial treatment. The primary site was located in the larynx in 11 (35.5%) and the hypopharynx in 20 (64.5%) patients. According to the 2002 AJCC staging system, all the patients had stage III/IV disease. Regarding the detailed tumour (T) distribution, 12 patients (38.7%) had T2 disease, with 8 tumours being hypopharyngeal and 4 transglottic. A total laryngectomy was considered necessary for successful disease control in these patients, since they had infiltrating tumours with a histologically aggressive nature. Thirteen (41.9%) and 6 (19.4%) patients had T3 and T4 tumours, respectively. Lymph node metastasis was detected in 26 (83.9%) patients, the majority of whom had N2 disease (22/26, 84.6%). The patients completed a minimum of two cycles of chemotherapy with the XP regimen and the mean number of completed cycles was 2.9±1.4 (range, 2–5).

### Treatment response

Following concurrent chemoradiotherapy, a clinical evaluation indicated that 23 (74.2%) and 7 (22.6%) patients exhibited CR and PR at the primary site, respectively, whereas 18 (69.2%) and 7 (26.9%) patients exhibited CR and PR at the lymph nodes, respectively. A CR at both the primary site and lymph nodes was identified in 19 patients (61.3%) (Table II).

Among the 8 patients who failed to achieve CR at the primary site after concurrent chemoradiotherapy, 3 patients underwent salvage laryngectomy and achieved successful local disease control. Two patients who had initial disease clearance after concurrent chemoradiotherapy later developed local recurrence during follow-up. Of the 2 patients, 1 underwent salvage laryngectomy; however, the tumour subsequently relapsed elsewhere.

Among the 8 patients who failed to achieve CR at the lymph nodes following concurrent chemoradiotherapy, 2 patients underwent neck dissection with resultant regional disease control. However, lung metastasis developed in these 2 patients during follow-up.

### Survival and laryngeal preservation

**Figure 1:**
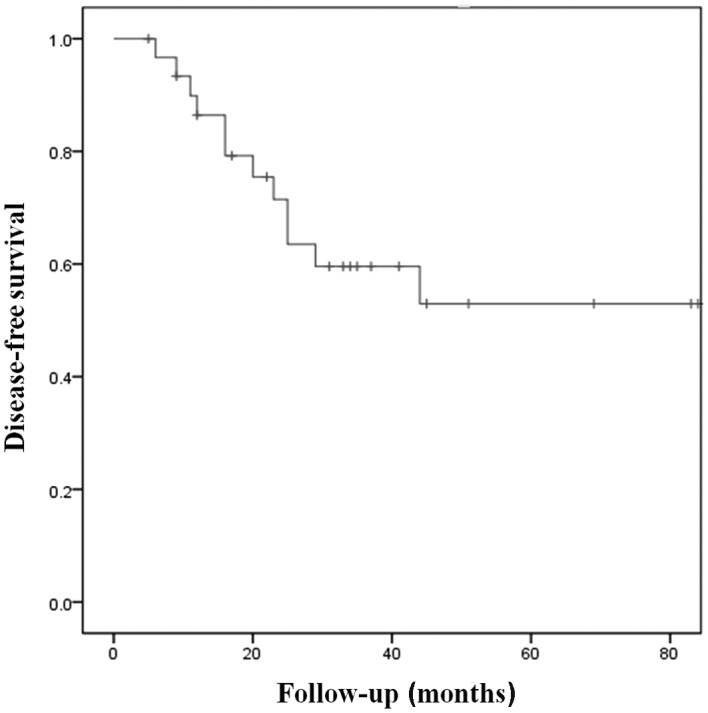
Disease-free survival curve for 31 patients with laryngeal-hypopharyngeal squamous cell carcinoma after concurrent chemoradiotherapy with capecitabine/cisplatin.

A total of 31 patients were followed-up for a mean of 35.9 months from the date of initial treatment. At the time of analysis, 19 patients (61.3%) remained alive and disease-free (Table III). No patient with disease remained alive. Of the 12 patients (38.7%) who succumbed to the disease, 10 deaths were LHSCC-related and 1 was due to a second primary malignancy. One patient died without evidence of head and neck cancer. Of the 19 patients who achieved CR at both the primary site and neck following concurrent chemoradiotherapy, 4 (21.1%) died. Among the non-CR patients, the 8 patients who failed to achieve CR at the lymph nodes eventually succumbed due to disease progression or recurrence regardless of the treatment response at the primary site or salvage surgery. The Kaplan-Meier 2- and 3-year cumulative disease-free survival rates were 71.5 and 59.6%, respectively (Fig. 1).

**Figure 2:**
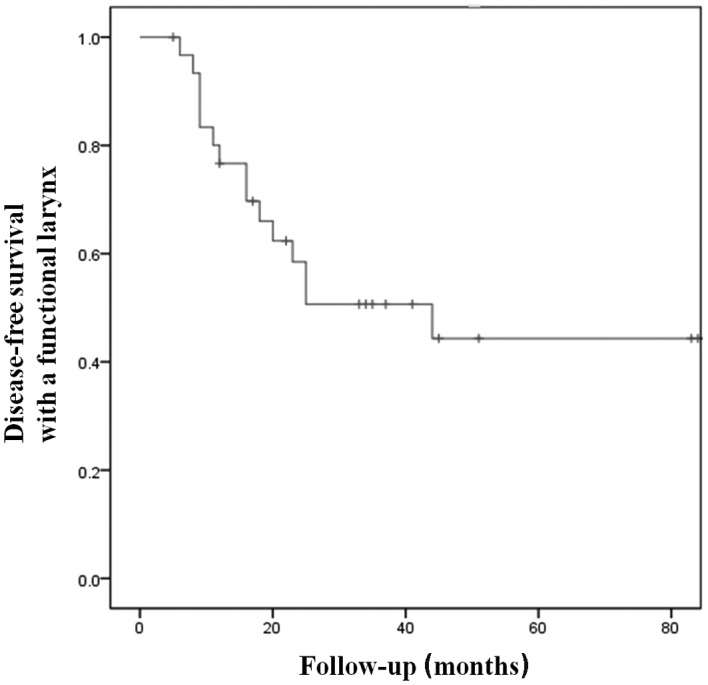
Disease-free survival with a functional larynx.

Anatomical organ preservation was possible in all but 4 of the 31 patients (12.9%) during the follow-up. Permanent gastrostomy was required in 1 disease-free patient with an intact larynx. At the time of the evaluation, 16 patients were alive without evidence of disease and with a functional larynx. For the entire 31-patient cohort, the 2- and 3-year cumulative disease-free survival with a functional larynx were 58.5 and 50.7%, respectively (Fig. 2).

### Pattern of treatment failure

Following concurrent chemoradiotherapy, overall treatment failure, including non-CR plus recurrence, was observed in 17 of the 31 patients (54.8%). During follow-up, local, regional and distant failure was observed in 10 (32.3%), 11 (35.5%) and 5 (16.1%) patients, respectively. Regional failure most commonly occurred at level III or IV compartment of the neck (8 of the 11 patients). Of the 5 patients who developed distant metastases, 2 presented with lung, 2 with liver and 1 with lung and bone metastasis. The 2- and 3-year cumulative local failure rates were 29.0 and 34.9%, respectively. The 2- and 3-year cumulative regional failure rate was 37.5%; all of the regional recurrence occurred within 2 years after completion of concurrent chemoradiotherapy. The 2- and 3-year cumulative distant failure rates were 17.5 and 24.4%, respectively. Second primary cancers were detected in 2 (6.5%) patients, in whom the primary site was the hypopharynx and the second primary cancer was oesophageal cancer.

### Toxicity

The haematological and non-haematological toxicities that occurred during concurrent chemoradiotherapy are summarised in Table IV. The most frequent haematological adverse events were anaemia and leucopenia, which occurred in 71.0% of the patients. Neutropenia and thrombocytopenia were observed in 64.5 and 6.4% of the patients, respectively. The most frequent grade 3–4 haematological toxicity was leucopenia; however, it only developed in 4 patients (12.9%). Regarding the non-haematological toxicities, mucositis was the one most frequently observed, with an overall incidence of 71.0%, followed by nausea with 61.3%. Grade 3–4 mucositis and nausea were observed in 25.8 and 16.1% of the patients, respectively. There was no treatment-related mortality identified in the present study.

## Discussion

Over the last two decades, larynx preservation has been considered one of the most important achievements in head and neck oncology. One of the second-generation clinical trials on laryngeal preservation, the RTOG 91-11 trial, which included intergroup trials of induction chemotherapy followed by irradiation, concurrent chemoradiotherapy and radiotherapy alone, verified that total laryngectomy may be avoided in numerous patients without jeopardising the chances of cure (4,5).

The primary endpoint of the present study was defined as disease-free survival with a functional larynx. This concept included the following conditions: survival; disease-free status at all sites, including primary site, regional lymph nodes and/or distant organs; and the retention of a functional larynx. The larynx preservation literature is replete with confusing and variably defined outcomes (5). Laryngeal preservation, for example, is defined in the RTOG 91-11 trial by the event of a laryngectomy (4). This suggests that patients who died with an intact larynx, even with laryngeal recurrence of the disease, would still be censored in the outcomes analysis (5). Therefore, certain authors have suggested that local control without surgery is a more informative endpoint to assess the success of any non-operative local therapy (5). However, local control without surgery may also disregard the cases of regional or distant treatment failure and treatment-related mortality. Therefore, disease-free or disease-controlled survival with a functional larynx was considered a more reasonable endpoint to estimate the efficacy of a treatment modality.

The 3-year cumulative disease-free survival with a functional larynx in our study was found to be 50.7%. This shows that more than half of the patients may achieve a CR and maintain a disease-free survival status without any additional treatment after concurrent chemoradiotherapy during a 36-month follow-up. This finding is comparable to the results of previous studies on advanced laryngeal or hypopharyngeal squamous cell carcinoma, which reported laryngeal preservation rates of 40–60% (12–14). In the present study, however, the full doses of capecitabine and cisplatin were not administered. In the majority of the studies on chemotherapy with or without concomitant radiotherapy, 100 mg/m^2^ cisplatin was administered alone or concurrently with 1,000 mg/m^2^ 5-FU (4,5,13–15). Of note, there was no evidence of a dose-response relationship (high dose, 120 mg/m^2^ vs. low dose, 60 mg/m^2^) for cisplatin activity in patients with advanced squamous cell carcinoma of the head and neck in a previous randomised trial (16). Specifically, recent studies on concurrent chemoradiotherapy in patients with squamous cell carcinoma of the head and neck used low-dose cisplatin (60 mg/m^2^) and reported comparable treatment results, with relatively low complication rates (17–19). Although a phase I study stated the recommended dose for the XP regimen (100 mg/m^2^ cisplatin and 1,000 mg/m^2^ capecitabine), the cisplatin dose was reduced since, in our experience, the majority of the critical complications of concurrent chemoradiotherapy with cisplatin and capecitabine are associated with cisplatin rather than capecitabine (20).

In the RTOG 91-11 trial, which included 547 patients who received cisplatin and concomitant radiotherapy for advanced laryngeal cancer, the locoregional control rate was 69% and the overall survival rate at 5 years during actuarial follow-up was 54% for the concurrent chemoradiotherapy group. Better locoregional control resulting in better laryngeal preservation was achieved in the concurrent chemoradiotherapy arm compared to the sequential chemotherapy and radiotherapy or the radiotherapy alone arms (4,15). However, the concurrent regimen was associated with statistically significant severe toxic effects (4,15). In the European Organisation for Research and Treatment of Cancer 24954 study, a phase III randomised trial on larynx preservation, comparing sequential and alternating chemotherapy and radiotherapy in 450 patients, the 3-year survival with a functional larynx was 39.5–45.4% and the 3-year overall survival was 62.2–64.8% (15). These results were comparable to those of our study. However, considering the higher dose of cisplatin (100 mg/m^2^) used in their study, the results of the XP regimen with a reduced dose of cisplatin (60 mg/m^2^) in our study may represent an improved outcome due to the addition of capecitabine. Moreover, the rate of grade 3–4 haematological toxicities and grade 3–4 mucositis in the RTOG 91-11 study (47 and 43%, repectively) were considerably higher compared to those observed in the present study (4). The incidence of grade 3–4 haematological toxicities in our study (anaemia, 3.2% and leucopenia, 12.9%) was also significantly lower compared to that of previous studies on regimens including 5-FU, in which the incidence of grade 3–4 anaemia and leucopenia was 18–81% (6,21,22). Furthermore, compared to our previous study, a generally lower incidence of haematological toxicity was observed in the present study that was likely due to the reduced dose of cisplatin (1). Therefore, the cisplatin dose (60 mg/m^2^) used in our study retained its efficacy, making it applicable for therapeutic regimens.

The favourable results obtained from treatment with capecitabine in combination with low-dose cisplatin may be explained mainly by its tumour specificity as determined by thymidine phosphorylase (TP), which is expressed at higher levels in tumour tissue compared to normal tissue (23). Furthermore, radiotherapy has been demonstrated to induce TP expression, and daily administration mimicking the continuous infusion of 5-FU can act as a radiosensitiser for every fraction of radiotherapy (1,24).

In our study, the 8 patients who failed to achieve CR at the lymph nodes following concurrent chemoradiotherapy succumbed due to disease progression or recurrence, whereas 4 of the 8 patients who failed to achieve CR at the primary site also died. It is well-known that the presence of lymph node metastasis decreases the survival rate by as much as 50%. This suggests that the disease control of only the primary site may not be sufficient unless metastatic lymph nodes are controlled. Therefore, the concept of disease control may be more informative compared to local control.

In a detailed analysis of 6 patients with T4 disease, the treatment results were not satisfactory: 3 patients succumbed due to treatment failure after concurrent chemoradiotherapy and 1 underwent total laryngectomy with partial pharyngectomy for local recurrence. In a recent study of 166 patients with stage III/IV laryngeal squamous cell carcinoma, cT4a patients treated with total laryngectomy exhibited a higher 3-year overall survival rate compared to patients treated with chemoradiotherapy (78 vs. 54%, respectively) (12). In addition, a smaller French randomised trial on advanced laryngeal cancer reported significantly improved survival for patients in the laryngectomy arm compared to those in the no-surgery experi mental arm (25). Therefore, the benefits of organ preservation and risks of treatment failure should be carefully weighed for patients with T4 LHSCC.

The main limitations of our study were the small patient sample and the insufficient follow-up period. Therefore, a more specific analysis according to the primary site or T stage could not be performed and the long-term results of laryngeal preservation could not be assessed. In addition, the effect of the convenience of oral administration of capecitabine on patient quality of life and treatment costs was not assessed.

In the present study, we demonstrated that concurrent chemoradiotherapy with the XP regimen resulted in significant functional laryngeal preservation with a satisfactory disease-free survival and toxicities that were more tolerable and manageable compared to those reported by previous studies (1,4,6). A large, prospective phase III study comparing the XP regimen to cisplatin alone or to the FP regimen, including assessments of quality of life and cost-effectiveness, is required to obtain additional information. Furthermore, additional long-term follow-up is required to evaluate delayed treatment results.

## Figures and Tables

**Table I:** Patient baseline demographics.

Variable	Patient no.^a^ (%)
Age, years (mean ± SD)	66.3±7.6
Gender	
Male	29 (93.5)
Female	2 (6.5)
ECOG PS	
0–1	25 (80.6)
2	6 (19.4)
Primary tumour site	
Larynx	11 (35.5)
Hypopharynx	20 (64.5)
Tumour distribution	
T2	12 (38.7)
T3	13 (41.9)
T4	6 (19.4)
Lymph node distribution	
N0	5 (16.1)
N1	4 (12.9)
N2a	4 (12.9)
N2b	11 (35.5)
N2c	7 (22.6)
No. of chemotherapy cycles (mean ± SD)	2.9±1.4

a Total patient no. was 31. SD, standard deviation; ECOG, Eastern Cooperative Oncology Group; PS, performance status.

**Table II:** Treatment response following concurrent chemoradiotherapy.

Variable	Patient no.^a^ (%)
Primary tumour (n=31)	
CR	23 (74.2)
PR	7 (22.6)
SD	1 (3.2)
Lymph node metastasis (n=26)	
CR	18 (69.2)
PR	7 (26.9)
SD	1 (3.9)
Primary tumour + lymph node (n=31)	
CR	19 (61.3)
PR	11 (35.5)
SD	1 (3.2)

a Total patient no. was 31. DFS, disease-free survival.

**Table III:** Survival and laryngeal preservation.

Variable	Patient no.^a^ (%)
Overall mortality	12 (38.7)
DFS	19 (61.3)
2-year cumulative	71.5
3-year cumulative	59.6
Laryngectomy	4 (12.9)
Non-functional larynx	1 (3.2)
DFS with a functional larynx	16 (51.6)
2-year cumulative	58.5
3-year cumulative	50.7

a Total patient no. was 31. CR, complete response; PR, partial response; SD, stable disease.

**Table IV:** Acute toxicities of concurrent chemoradiotherapy.

Toxicities	Grade [patient no. (%)]				
	1	2	3	4	3–4
Haematological (n,%)					
Anaemia	11 (35.5)	10 (32.3)	0 (0.0)	1 (3.2)	1 (3.2)
Leucopenia	4 (12.9)	14 (45.2)	3 (9.7)	1 (3.2)	4 (12.9)
Neutropenia	5 (16.1)	12 (38.7)	2 (6.5)	1 (3.2)	3 (9.7)
Thrombocytopenia	1 (3.2)	1 (3.2)	0 (0.0)	0 (0.0)	0 (0.0)
Non-haematological (n, %)					
Nausea	8 (25.8)	6 (19.4)	5 (16.1)	0 (0.0)	5 (16.1)
Vomiting	5 (16.1)	5 (16.1)	4 (12.9)	0 (0.0)	4 (12.9)
Mucositis	9 (29.0)	5 (16.1)	6 (19.4)	2 (6.5)	8 (25.8)
Diarrhoea	2 (6.5)	1 (3.2)	1 (3.2)	0 (0.0)	1 (3.2)
Hand-foot syndrome	5 (16.1)	2 (6.5)	0 (0.0)	0 (0.0)	0 (0.0)
